# Supplementary material for: Solanidine‐derived CYP2D6 phenotyping elucidates phenoconversion in multimedicated geriatric patients
Source: Br J Clin Pharmacol. 2025 Feb 16;91(6):1842–52. doi: 10.1002/bcp.70004 (PMC12272519; doi:10.1002/bcp.70004)
Supplement: Supplementary file 1 — SUPPORTING INFORMATION DATA S1 Definition of parameters used to calculate the Charlson Comorbidity Index and corresponding ICD‐10 codes SUPPORTING INFORMATION DATA S2 Synthesis of 4‐OH‐solanidine SUPPORTING INFORMATION DATA S3 Chromatograms of (A) 10 ng mL−1 solanidine before incubation with CYP2D6*1 supersomes, (B) 10 ng mL−1 solanidine after 24 h of incubation with CYP2D6*1 supersomes, (C) a representative plasma sample and (D) 0.25 ng mL−1 4‐OH‐solanidine standard SUPPORTING INFORMATION DATA S4 Solanidine and seco‐solanidine‐3,4‐dioic acid (SSDA) plasma levels as measured via liquid chromatography tandem mass spectroscopy in the cohort (N = 88). Genotype‐predicted phenotypes are shown in colour. Undetected levels are printed as the lowest detected area divided by two SUPPORTING INFORMATION DATA S5 Diplotypes of cytochrome P450 (CYP) 2D6 and respective genotype‐predicted phenotypes in the study population of geriatric, multimedicated patients (N = 88) SUPPORTING INFORMATION DATA S6 Natural logarithm of the metabolic ratios (ln MR) of (A) 3,4‐seco‐solanidine‐3,4‐dioic acid (SSDA)/solanidine (P = .019) and (B) 4‐OH‐solanidine/solanidine (P = .012) measured in a population of geriatric, multimedicated patients after exclusion of CYP2D6 poor metabolizers stratified to no vs any use of CYP2D6 substrates and inhibitors SUPPORTING INFORMATION DATA S7 Shift in AS based on (A) 3,4‐seco‐solanidine‐3,4‐dioic acid (SSDA)/solanidine (F = 2.874, P = .042) and (B) 4‐OH‐solanidine/solanidine (F = 2.165, P = .099) metabolic ratios in a population of geriatric, multimedicated patients after exclusion of CYP2D6 poor metabolizers stratified to four groups with and without use of CYP2D6 substrates and inhibitors SUPPORTING INFORMATION DATA S8 Linear regression of the shift in activity score vs the numbers of cytochrome P450 (CYP) 2D6 substrates and inhibitors documented in the patients' medication with the shift in AS calculated based on (A) seco‐solanidine‐3,4‐dioic acid (SSDA) measu [file BCP-91-1842-s001.docx]

**Supplement to:**

Solanidine-derived CYP2D6 phenotyping elucidates phenoconversion in multi-medicated geriatric patients

## Authors

Jens Andreas Sarömba^1^, Julian Peter Müller^1^, Jolanta Tupiec^1^, Anjali Roeth^2^, Berkan Kurt^3^, Florian Kahles^3^, Thea Laurentius^4,5^, Cornelius Bollheimer^4^, Julia C Stingl^1^, Katja S Just^1^

Corresponding Author: Katja S Just: kjust@ukaachen.de

## Author Affiliations

1 Institute of Clinical Pharmacology, University Hospital RWTH Aachen

2 Department of General, Visceral, Pediatric and Transplantation Surgery, University Hospital RWTH Aachen

3 Department of Internal Medicine I - Cardiology, University Hospital RWTH Aachen

4 Department of Geriatric Medicine, University Hospital RWTH Aachen

5 Department of Geriatrics, Carl von Ossietzky University of Oldenburg

**Supplement 1**: Definition of parameters used to calculate the Charlson Comorbidity Index and corresponding ICD-10 codes

| **Disease** | **ICD 10 code** | **Points** |
| --- | --- | --- |
| Diabetes, uncomplicated | E10, E11, E12, E13, E14 | 1 |
| Myocardial infarction | I21, I25 | 1 |
| Heart failure | I50 | 1 |
| Peripheral artery disease | I70 | 1 |
| Stroke or transient ischemic attacks | I63, I64, G45 | 1 |
| Dementia | F0, F01, F02, F03 | 1 |
| Chronic obstructive pulmonary disease | J44 | 1 |
| Gastritis or peptic ulcer disease | K2 | 1 |
| Liver disease, mild | K7 | 1 |
| Hemiplegia or hemiparesis | G81 | 2 |
| Tumor, leukemia, lymphoma | Any C code | 2 |

ICD-10: International Statistical Classification of Diseases and Related Health Problems, Version 10. Connective tissue diseases, acquired immune deficiency syndrome (AIDS), and non-mild liver disease were not documented in the cohort.

**Supplement 2:** Synthesis of 4-OH-solanidine

**Supplement 2:** Synthesis of 4-OH-solanidine by oxidizing solanidine with selenium dioxide in dioxane in the presence of formic acid and heating.


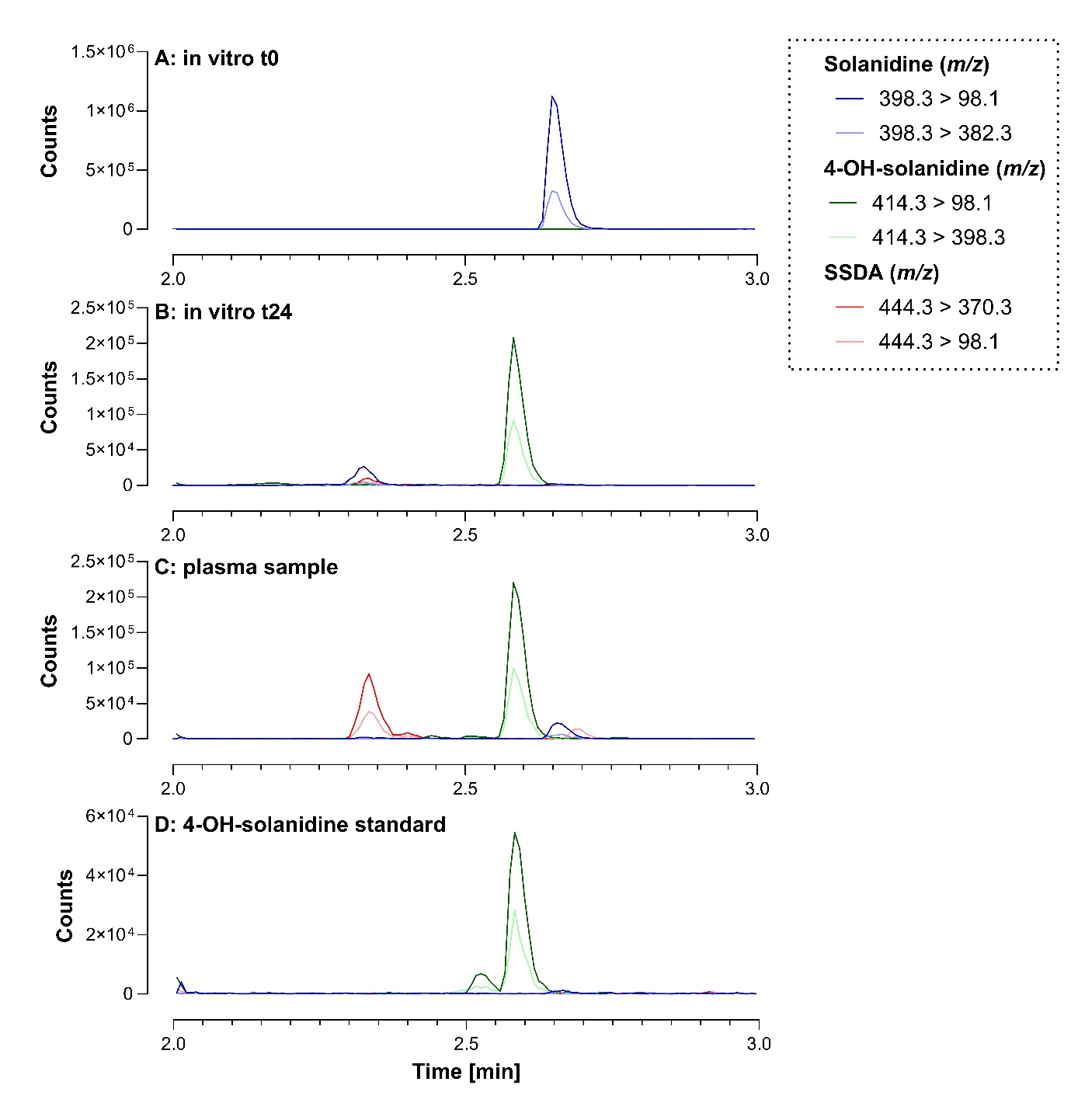


**Supplement 3**: Chromatograms of A) 10 ng ml^-1^ solanidine before incubation with CYP2D6*1 supersomes B) 10 ng ml^-1^ solanidine after 24 h incubation with CYP2D6*1 supersomes C) a representative plasma sample D) 0.25 ng ml^-1^ 4-OH-solanidine standard

CYP2D6: Cytochrome P450 2D6, SSDA: 3,4-seco-solanidine-3,4-dioic acid, m/z: mass-to-charge ratio


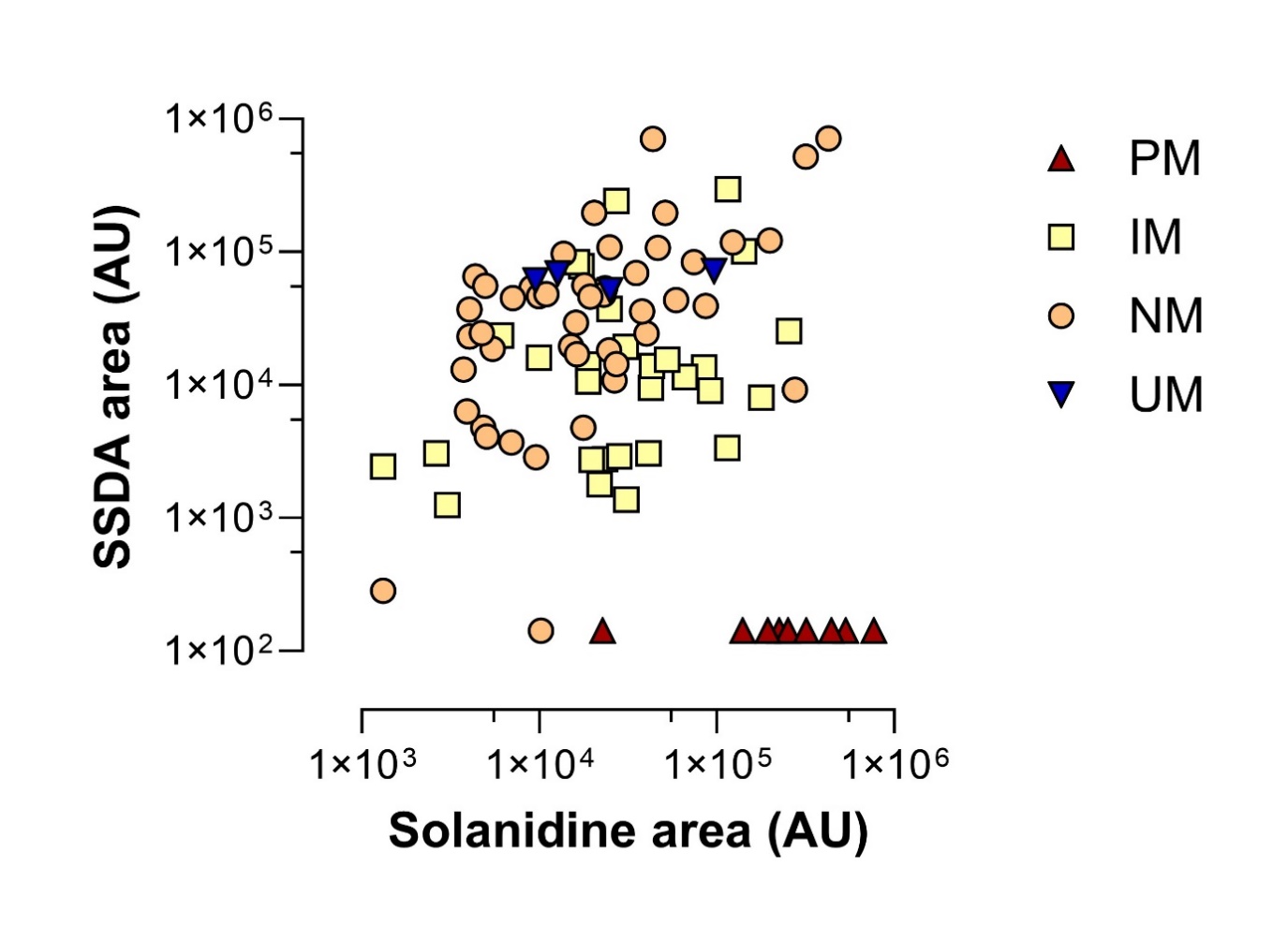


**Supplement 4:** Solanidine and SSDA plasma levels as measured via liquid chromatography tandem mass spectroscopy (LC-MS/MS) in the cohort (N=88). In color, genotype-predicted phenotypes are shown. Undetected levels are printed as the lowest detected area divided by two.

PM: Poor metabolizer, IM: Intermediate metabolizer, NM: Normal metabolizer, UM: Ultra rapid metabolizer, SSDA: 3,4-seco-solanidine-3,4-dioic acid, AU: Arbitrary units

**Supplement 5**: Diplotypes of cytochrome P450 (CYP) 2D6 and respective genotype-predicted phenotypes in the study population of geriatric, multi-medicated patients (N=88).

| **Predicted phenotype** | **Assigned activity score** | **Diplotypes** | **Frequency, n (%)** |
| --- | --- | --- | --- |
| Poor metabolizer (PM) | 0 | *4/*4  *4/*5 | 4 (4.5)  5 (5.7) |
| Intermediate metabolizer (IM) | 0.25 | *4/*10  *4/*41  *5/*41 | 2 (2.3)  3 (3.4)  1 (1.1) |
|  | 1 | *1/*4  *1/*5  *1/*6  *2/*3 | 17 (19.3)  4 (4.5)  1 (1.1)  1 (1.1) |
| Normal metabolizer (NM) | 1.25 | *1/*10  *1/*41  *1/*9  *2/*9  *2/*10  *2/*41 | 4 (4.5)  3 (3.4)  1 (1.1)  1 (1.1)  1 (1.1)  3 (3.4) |
|  | 2 | *1/*1  *1/*2  *2/*2 | 8 (9.1)  18 (20.5)  7 (8.0) |
| Ultra rapid metabolizer (UM) | 3 | *1/*1x2  *1/*2x2  *2/*2x2 | 1 (1.1)  1 (1.1)  2 (2.3) |

**
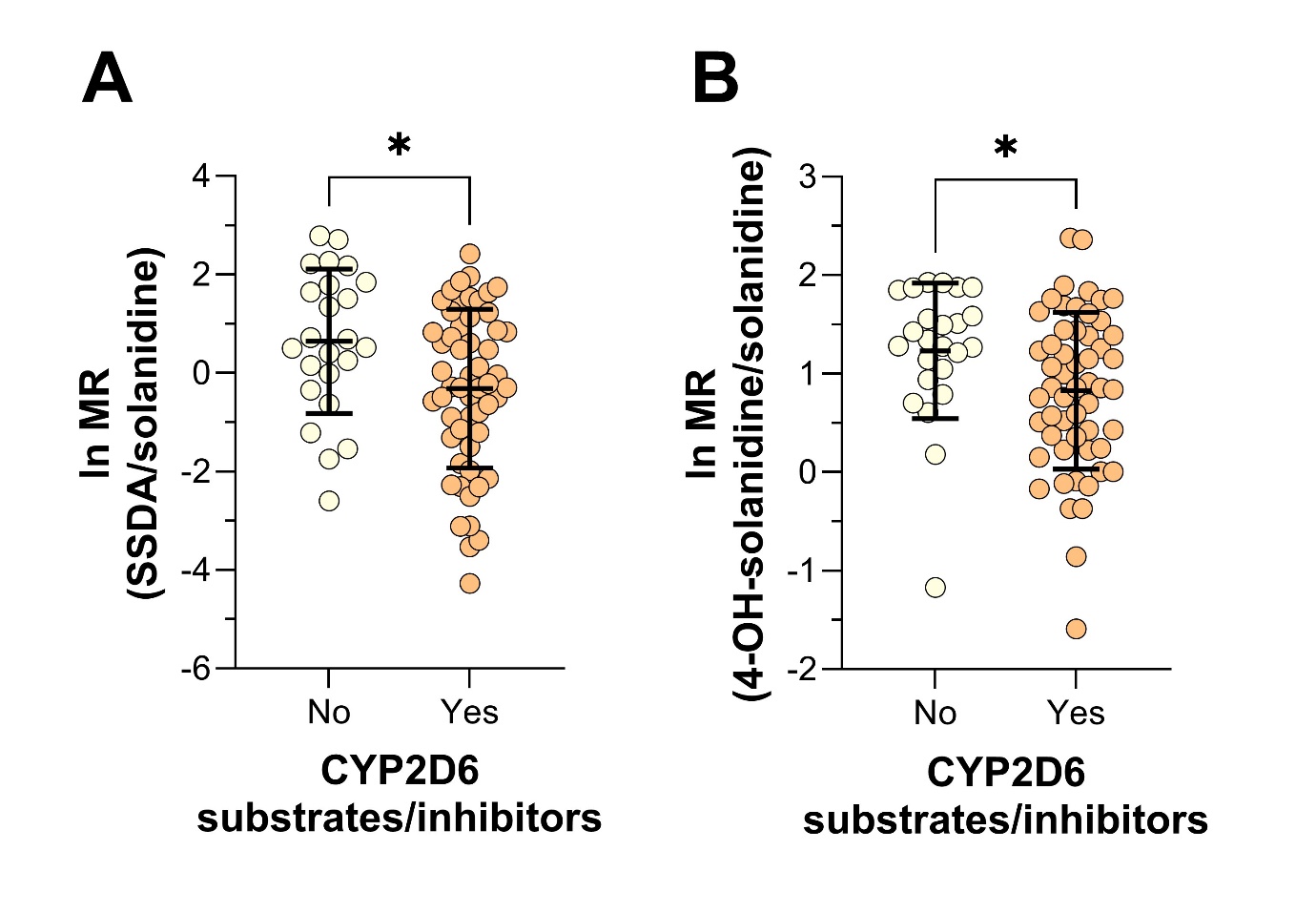
**

**Supplement 6**: Natural logarithm of the metabolic ratios (ln MR) of **A)** 3,4-seco-solanidine-3,4-dioic acid (SSDA)/solanidine (p=0.019) and **B)** 4-OH-solanidine/solanidine (p=0.012) measured in a population of geriatric, multi-medicated patients after exclusion of CYP2D6 poor metabolizers stratified to no versus any use of CYP2D6 substrates and inhibitors.


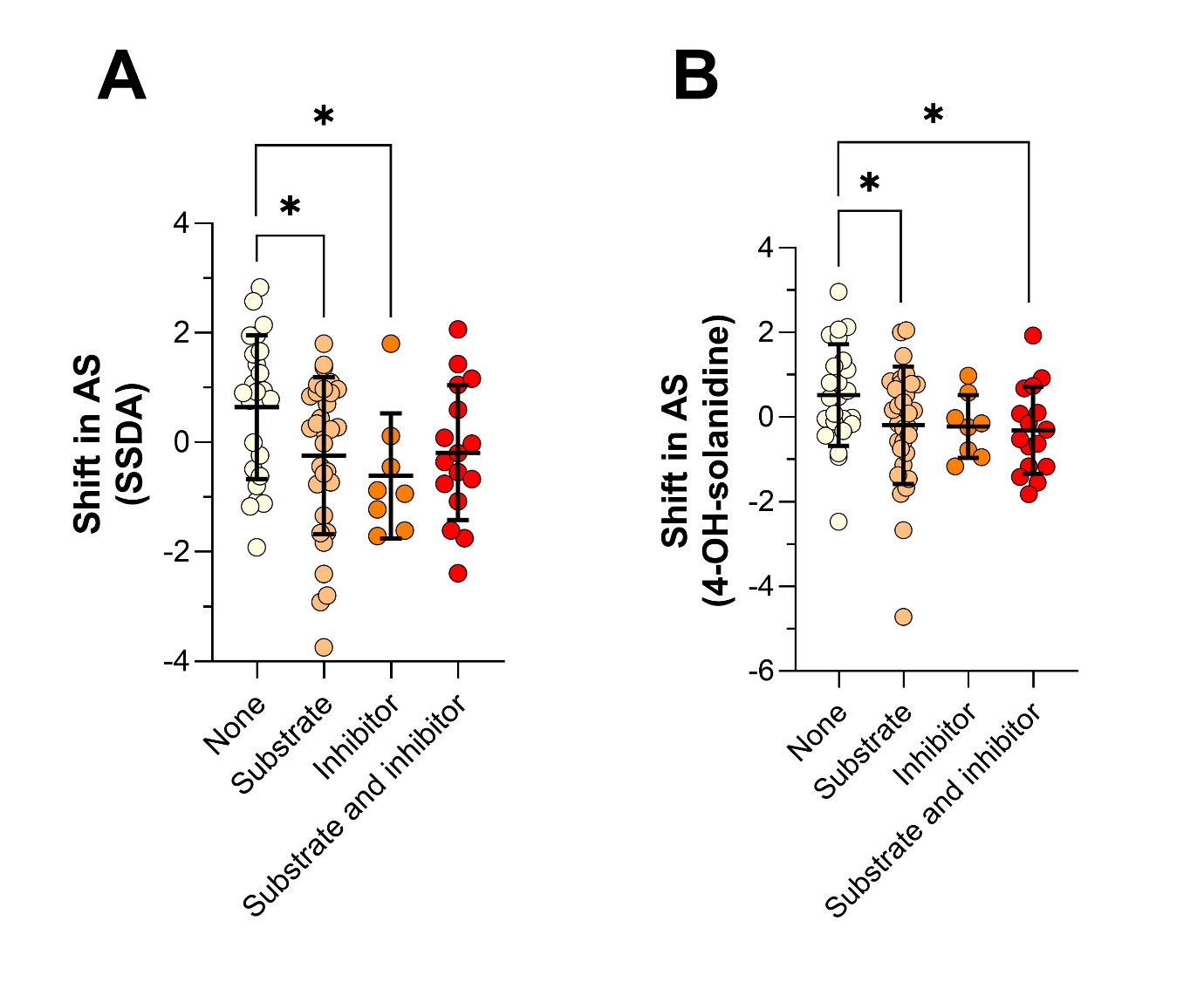


**Supplement 7**: Shift in AS based on **A)** 3,4-seco-solanidine-3,4-dioic acid (SSDA)/solanidine (F= 2.874, p=0.042) and **B)** 4-OH-solanidine/solanidine (F=2.165, p=0.099) metabolic ratios in a population of geriatric, multi-medicated patients after exclusion of CYP2D6 poor metabolizers stratified to four groups with and without use of CYP2D6 substrates and inhibitors.

Comparisons were made using one-way ANOVA with post-hoc one-sided Dunnett’s test assuming patients without any use of substrates or inhibitors to have a higher shift in AS. To compare the shift in AS based on SSDA/solanidine and 4-OH-solanidine/solanidine metabolic ratios between the group without use of substrates and inhibitors (None) with the following three groups: with use of substrates and without inhibitors (Substrate, A) p=0.023, B) p=0.046), without use of substrates and with use of inhibitors (Inhibitor, A) p=0.32, B) p=0.168), and with the use of substrates and inhibitors (Substrate and inhibitor, A) p=0.075, B) p=0.049).


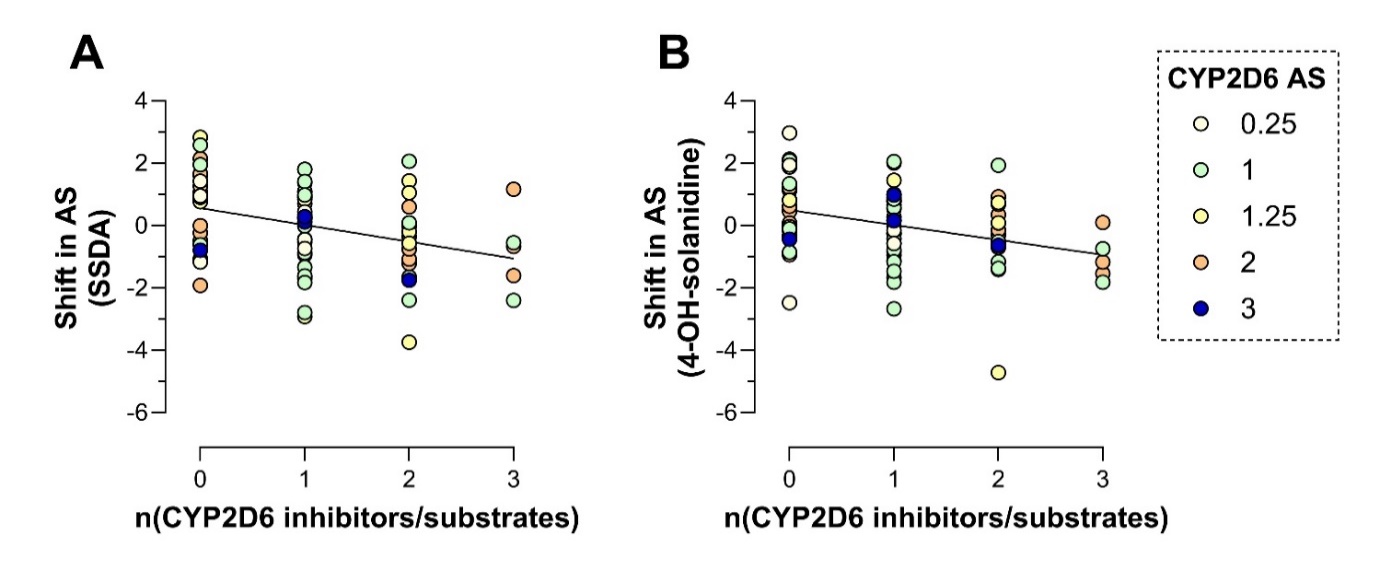


**Supplement 8:** Linear regression of the shift in activity score versus the numbers of cytochrome P450 (CYP) 2D6 substrates and inhibitors documented in the patients’ medication with the shift in AS calculated based on **A)** SSDA measurements (p=0.002, R^2^=0.119) and **B)** 4-OH-solanidine measurements (p=0.002, R^2^=0.117).

Coloring is based on CYP2D6 activity scores.

AS: Activity score, SSDA: 3,4-seco-solanidine-3,4-dioic acid


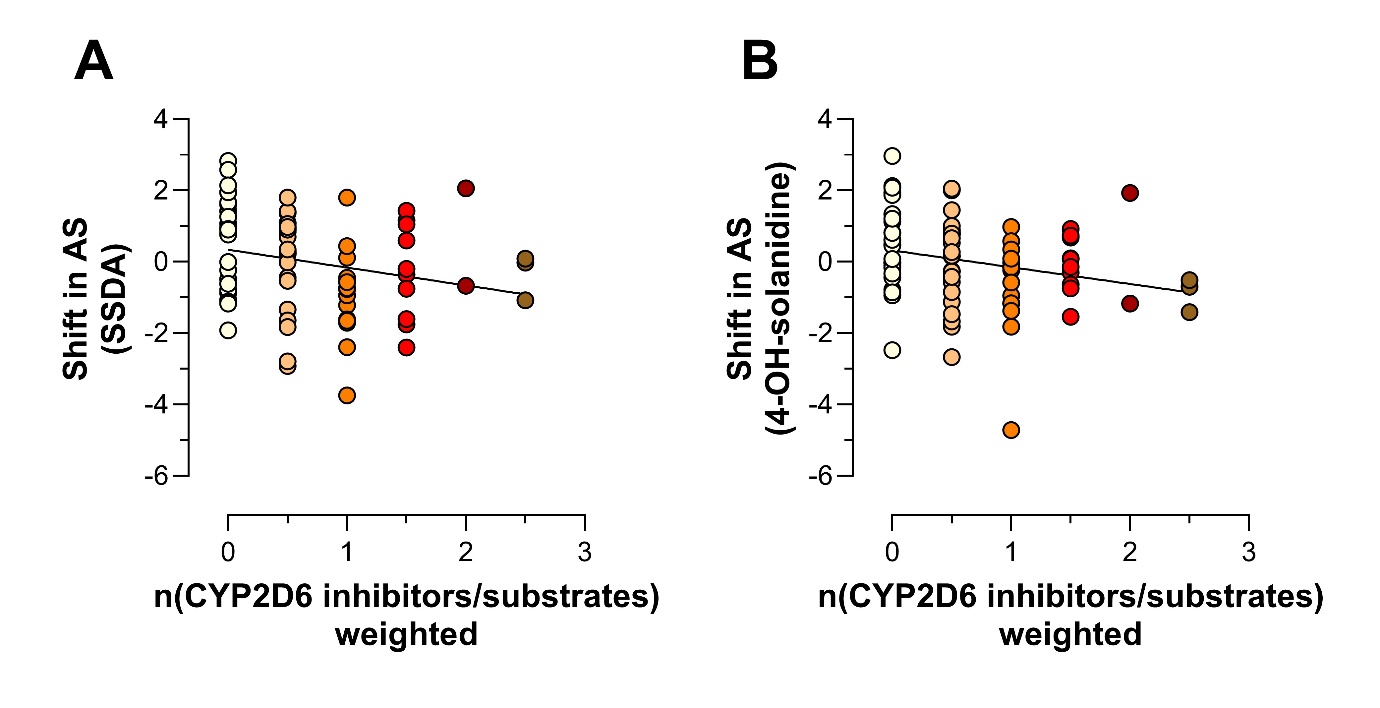


**Supplement 9**: Linear regression of the shift in activity score (AS) versus the numbers of cytochrome P450 (CYP) 2D6 substrates and inhibitors documented in the patients’ medication using a weighted inhibition score. Calculation of shift in AS based on **A)** 3,4-seco-solanidine-3,4-dioic acid (SSDA) measurements (p=0.033, R^2^=0.057) and **B)** 4-OH-solanidine measurements (p=0.026, R^2^=0.063).

The following weights were used: substrate: 0.5, weak inhibitor, unclear inhibitor, in vitro evidence only inhibitor: 1, moderate inhibitor: 2.
